# Artificial Intelligence‐Augmented Pediatric Lung POCUS: A Pilot Study of Novice Learners

**DOI:** 10.1002/jum.15992

**Published:** 2022-04-15

**Authors:** Benjamin Nti, Amalia S. Lehmann, Aida Haddad, Sarah K. Kennedy, Frances M. Russell

**Affiliations:** ^1^ Division of Pediatric Education, Department of Pediatrics Indiana University School of Medicine Indianapolis IN USA; ^2^ Department of Emergency Medicine, Department of Pediatrics Indiana University School of Medicine Indianapolis IN USA

**Keywords:** artificial intelligence, lung, pediatrics, ultrasound

## Abstract

**Objective:**

Respiratory symptoms are among the most common chief complaints of pediatric patients in the emergency department (ED). Point‐of‐care ultrasound (POCUS) outperforms conventional chest X‐ray and is user‐dependent, which can be challenging to novice ultrasound (US) users. We introduce a novel concept using artificial intelligence (AI)‐enhanced pleural sweep to generate complete panoramic views of the lungs, and then assess its accuracy among novice learners (NLs) to identify pneumonia.

**Methods:**

Previously healthy 0‐ to 17‐year‐old patients presenting to a pediatric ED with cardiopulmonary chief complaint were recruited. NLs received a 1‐hour training on traditional lung POCUS and the AI‐assisted software. Two POCUS‐trained experts interpreted the images, which served as the criterion standard. Both expert and learner groups were blinded to each other's interpretation, patient data, and outcomes. Kappa was used to determine agreement between POCUS expert interpretations.

**Results:**

Seven NLs, with limited to no prior POCUS experience, completed examinations on 32 patients. The average patient age was 5.53 years (±1.07). The median scan time of 7 minutes (minimum–maximum 3–43; interquartile 8). Three (8.8%) patients were diagnosed with pneumonia by criterion standard. Sensitivity, specificity, and accuracy for NLs AI‐augmented interpretation were 66.7% (confidence interval [CI] 9.4–99.1%), 96.5% (CI 82.2–99.9%), and 93.7% (CI 79.1–99.2%). The average image quality rating was 2.94 (±0.16) out of 5 across all lung fields. Interrater reliability between expert sonographers was high with a kappa coefficient of 0.8.

**Conclusion:**

This study shows that AI‐augmented lung US for diagnosing pneumonia has the potential to increase accuracy and efficiency.

**Abbreviations**
AI: artificial intelligence
AL: anterior lung
CI: confidence level
CXR: chest radiography
ED: emergency department
LL: lateral lung
LR: likelihood ratio
NL: novice learner
PL: posterior lung
POCUS: point‐of‐care ultrasound
SEM: standard error of mean
US: ultrasound

Respiratory symptoms are one of the most common chief complaints in the pediatric emergency department (ED), and pneumonia continues to be a leading infectious cause of death in children worldwide.^1^, ^2^ Despite guidelines supporting the diagnosis of pneumonia clinically, the use of chest radiography (CXR) has become the standard evaluation tool and is often overused leading to increased length of stay, radiation exposure, and expense.^3^, ^4^ Additionally, there is high inter‐ and intraobserver interpretation variability due to differing radiologic findings, which affects the sensitivity and specificity of this modality.^4^, ^5^

The use of pulmonary or lung point‐of‐care ultrasound (POCUS) has become a reliable tool for diagnosing patients presenting to the ED with a respiratory complaint.^6^ However, lung POCUS for the diagnosis of pneumonia can be challenging for novice learners (NLs) as interpretation fundamentally relies on understanding artifacts, rather than anatomical structures for diagnostic recognition.^7^ Furthermore, the standard approach to integrating lung POCUS described previously,^8^ is more favorable for experienced sonographers as they have higher diagnostic accuracy than novice sonographers.^9^ However, it is possible that with the emergence of new innovations such as artificial intelligence (AI) technology, which can streamline POCUS interpretation more accurately with greater efficiency, allowing POCUS to be more easily integrated into emergency management.^10^ To this regard, AI has been shown to have the ability to distinguish fluid responsive versus fluid unresponsive septic shock in the emergent setting with moderate agreement.^11^ AI‐assisted lung POCUS has also shown promise in diagnosing pulmonary infiltrates indicative of pneumonia in the pediatric inpatient setting with 100% specificity and 90.9% sensitivity.^12^ Additionally, AI has been shown to guide novice users to acquire high‐quality bedside cardiac ultrasound (US) images.^8^ This is also in line with prior studies that demonstrated that AI algorithm can guide novices without prior ultrasonography experience to acquire images for evaluation of left ventricular size and non‐trivial pericardial effusion.^13^

An inherent challenge of lung POCUS includes obtaining a complete pleural profile of lung parenchyma. As such, current standard protocols involve multiple windows with the same probe orientation in order to evaluate the anatomy completely. This can often lead to increased time required for image acquisition, leading to delay in medical decision making and patient disposition.^9^ This study introduces a novel concept using AI‐enhanced pleural sweep to generate complete panoramic windows for clinical evaluation by NLs. This study explores the role of AI in the future of lung POCUS where machine‐assisted image acquisition and interpretation can augment efficient and accurate clinical management by NL in the ED.

## Methods

### Study Setting and Population

This was a prospective study of pediatric patients aged 0 to 17 years presenting with a cardiopulmonary chief complaint between September 2018 and December 2019. Patients were enrolled from one urban academic quaternary pediatric hospital ED. We included patients presenting with respiratory complaints such as cough, shortness of breath, and fever reflecting patients with pneumonia as part of their differential diagnosis and workup. Potential participants were excluded from the study if they were not English or Spanish speaking, had a pre‐arrival diagnosis of pneumonia, or known cardiopulmonary disease including congenital heart disease, chronic lung disease, bronchopulmonary dysplasia, cystic fibrosis, known malignancy‐related pulmonary manifestations, or history of thoracic surgery. This study was approved by the local Institutional Review Board. Parental consent was obtained for all participants, and assent was obtained for patients greater than 7 years of age.

### Study Protocol

NLs included general Emergency Medicine residents, categorical Pediatric residents, and Pediatric Emergency Medicine fellows with limited to no prior general or lung‐specific POCUS experience (≤10 previously performed lung US) (Table 1). NLs completed a survey questionnaire assessing their prior experience with lung POCUS. They received a 1‐hour lecture and hands‐on training in lung POCUS. The lecture covered an introduction to traditional lung POCUS scan consisting of the 8‐zone evaluation as described previously and recognition of various lung pathology including pneumonia, effusion, edema, pneumothorax, subpleural consolidations, and contusions.^14^ The learners were then introduced to the AI software with image acquisition and interpretation. Immediately following the lecture, learners performed supervised scanning in the traditional lung POCUS views and then subsequently practiced using the AI image acquisition software on healthy standardized patients.

**Table 1:** Training Level at Start of Study and Experience of the Seven NL Participants

Novice Participant					
	Training	Level	Prior Total US	Prior Lung POCUS	Number Enrolled
Novice #1	PGY4	PEM fellow	>100	1–5	5
Novice #2	PGY4	PEM fellow	51–100	6–10	7
Novice #3	PGY1	EM intern	26–50	1–5	3
Novice #4	PGY1	EM intern	26–50	6–10	2
Novice #5	PGY1	EM intern	1–5	1–5	2
Novice #6	PGY2	Pediatric resident	1–5	0	9
Novice #7	PGY1	EM intern	0	0	4

PEM, pediatric emergency medicine; EM intern, first training year general emergency medicine; PGY, postgraduate year.

#### Software, Hardware, and Image Acquisition Protocol

**Figure 1:**
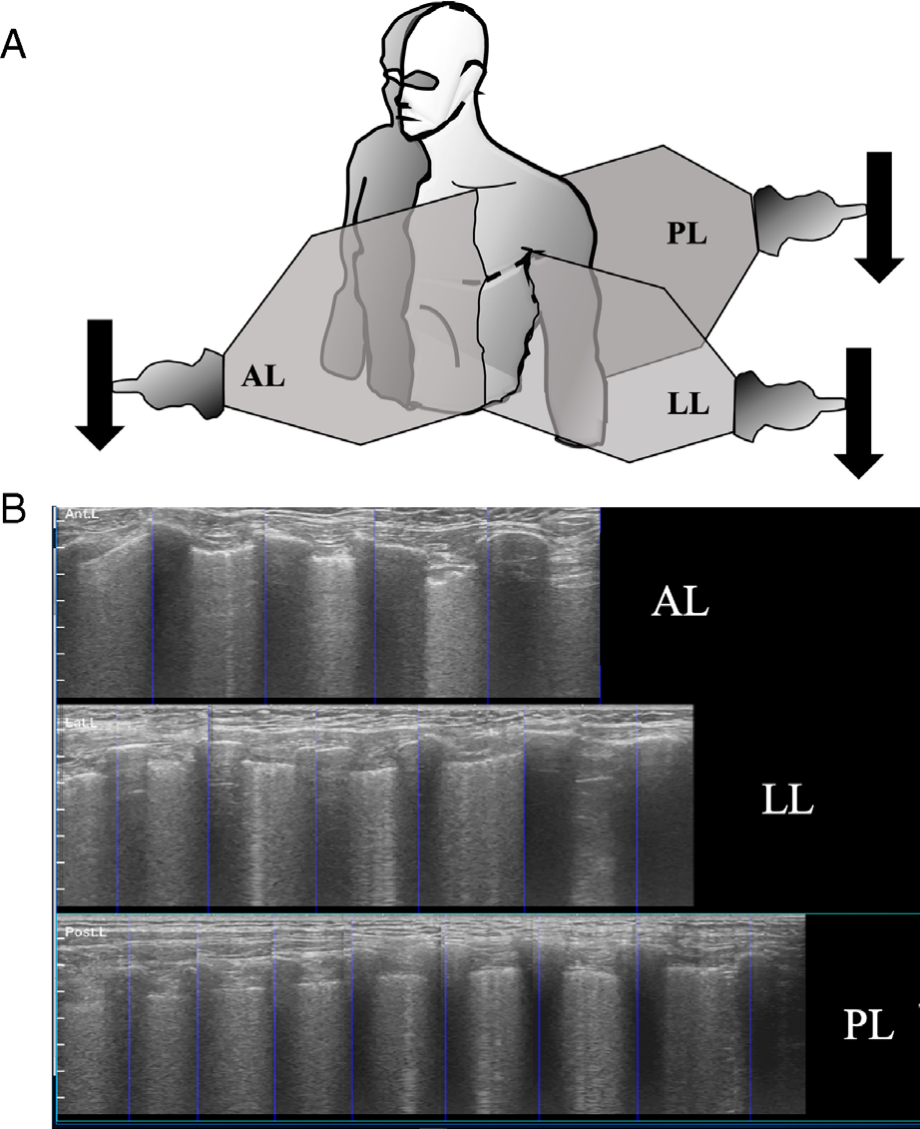
**A**, Unilateral illustration of the lung POCUS sweep caudally in the AL, PL, and lateral lung direction. **B**, Panoramic output of the lung sweep with each zone and orientation represented. Note extended views of the linear pleura from left (cephalad) to right (caudad) captured on the machine–AI capture of hypoechoic rib shadows in plane.

Imaging acquisition was based on the standard Bedside Lung Ultrasound in Emergency (BLUE) protocol, which features an 8‐zone approach.^15^ The probe was oriented in a primarily sagittal plane with the probe indicator facing the patient's head and the probe face perpendicular to the adjacent ribs. A continuous sagittal sweep was performed in three anatomic planes of the thorax bilaterally with soundwave projection directed as follows: anterior lung (AL) with sweep from midclavicular to costal margin; lateral lung (LL) with sweep from midaxillary to the superior aspect of the hepato‐diaphragmatic position; and posterior lung (PL) beginning just medial to scapula at the level of first intercostal space to the costal margin; this resulted in six images obtained per examination (Figure 1). The AI software required continuous contact of each hemithorax in the anatomic planes described previously (online supplemental Video 1). All scans were performed in real time using a general electric prototype Lung Sweep software on a laptop connected to an US machine (Mindray Zonare ZS3) with a linear transducer (L10). In brief, the software utilizes a series of algorithms to determine features within the images to provide assistance with anatomical orientation, quality, and speed of image acquisition without providing an interpretation in real time. The user was able to rescan if a clip was inadequate according to the software or the NLs assessment of image quality. The length of each clip was approximately 3 to 6 seconds depending on patient size, plane of the sweep, and real‐time software feedback on the appropriate velocity with the highest image quality. The software assisted the NL with each image acquisition allowing the learner to interpret the images at a later time. After the AI‐assisted lung POCUS was performed by NLs, they completed a qualitative assessment and interpreted the images to determine whether the there was evidence for pneumonia, indeterminant finding, or negative for pneumonia using a standardized assessment form. The NL did not perform a traditional lung POCUS prior to completing the AI‐assisted evaluation. It is important to note that the AI‐assisted lung POCUS evaluation was not used for patient care in this study and therefore, interpretation of acquired images was not completed in real time. Two fellowship‐trained POCUS experts with more than 15 years and greater than 1500 scans combined experience in lung US reviewed POCUS images obtained by the NLs. Each expert reviewed 50% of randomly selected NLs acquired images and interpretation. The expert's interpretations served as the criterion standard. This included assessment of the images for evidence of pneumonia, indeterminant finding, or negative for pneumonia. They were blinded to NLs' AI‐augmented interpretation and patient data. The POCUS experts assessed for lung sliding, A‐lines, B‐lines, consolidations, subpleural thickening, and pleural effusions. Interpretation of pneumonia on lung POCUS was defined as greater than two focal B‐lines (discrete vertical hyperechoic artifacts) in the presence or absence of a subpleural hypoechoic mass defect as described previously by Lichtenstein et al 2014.^7^, ^8^, ^15^

### Outcome Measures

NLs were blinded to the clinical workup and to patient outcomes. Using expert interpretation of NL obtained images as the criterion standard, we assessed the accuracy of AI‐assisted NL interpretation, calculating sensitivity, specificity, and likelihood ratios (LRs). Experts graded the quality of images using a rating scale from 1 to 5 (1 = no recognizable structures, no objective data can be gathered; 2 = minimally recognizable structures but insufficient for diagnosis; 3 = minimal criteria met for diagnosis, recognizable structures but with some technical or other flaws; 4 = minimal criteria met for diagnosis, all structures imaged well and diagnosis easily supported; 5 = minimal criteria met for diagnosis, all structures imaged with excellent image quality and diagnosis completely supported). For further assessment of NL image acquisition, ratings score of 1 to 2 was considered below average, while a score of 3 was designated as an average score. Above‐average rating scores were 4 or greater.

### Data Analysis

All data were collected and entered into a secure RedCap database, and images were securely stored in a Box storage (Box, Inc, Redwood City, CA) server or securely, wirelessly uploaded to an imaging acquisition software. A minimum sample of 30 was calculated using a confidence level (CI) of 95% and an alpha of 5% assuming a target population 100, which was based on 10% of actual population of estimated patients with pneumonia. Descriptive summary statistics were generated for demographic data. For Cohen's kappa analysis (ƙ), each expert reviewed one‐third (10 images) of the other's assigned images. Discrepancies were resolved by a third expert who was blinded to the NLs' AI‐augmented interpretation and the two expert reviewer's assessments.

## Results

This study recruited 32 patients with a mean age of 5.5 years (standard error of mean [SEM] ±1.1). Patients were predominantly male and Caucasian (Table 2). Clinical discharge diagnoses (35) are shown in Table 3, of which 3 (8.8%) were pneumonia. NL interpreted three patients with pneumonia, with two true positive finding and one assessed to be indeterminate but subsequently interpreted negative by experts' evaluation. All but one NL interpretation was determined to be true negative. Of the findings listed, three of the participants were discharged with multiple finding diagnoses. Seven total NLs completed the POCUS examinations. Twenty (63%) patients received a CXR as part of their workup, independent of the study evaluation. Even though this imaging test is inferior to US for detection of pneumonia, two patients were determined to be positive, consistent with NLs' AI‐augmented and expert interpretation. At the start of study recruitment, NLs were in postgraduate training years 1 to 4 (PGY1–4) of their respective training programs, which included emergency medicine residency (*n* = 4, 57%), pediatric residency (*n* = 1, 14%), and pediatric emergency medicine fellowship (*n* = 2, 29%) (Table 1). They completed an average of 4.71 patient scans each with a median image acquisition time of 7 minutes (Table 4).

**Table 2:** Demographics of Study Patients

Participant Demography		*n* = 32
Total participants	Description	32 (100%)
Gender	Female	12 (37.5%)
	Male	20 (62.5%)
Race	White	18 (56.2%)
	Black or African American	8 (25.0%)
	White and Black or African American	3 (9.3%)
	Declined	2 (6.2%)
Ethnicity	Hispanic or Latino	8 (25.0%)
	No Hispanic or Latino	23 (71.8%)
Ages (years)	0–5	20 (62.5%)
	6–10	3 (9.3%)
	11–15	7 (21.8%)
	16–18	2 (6.2%)
Average age (SEM)	Average = 5.53 (±1.07)	

**Table 3:** Discharge Diagnoses of Study Patients Which a Predominant Viral Syndrome Disposition With a Small Percentage Diagnosed With Pneumonia

Discharge Diagnoses	*n* = 35
Viral syndrome/acute URI/viral URI	10 (29.4%)
Asthma exacerbation	5 (14.3%)
Bronchiolitis	4 (11.4%)
Cough	4 (11.4%)
Pneumonia	3 (8.8%)
Chest pain	2 (5.8%)
Fever	2 (5.8%)
Abdominal pain	1 (2.9%)
Atypical pneumonia vs bronchitis	1 (2.9%)
GERD	1 (2.9%)
Group A strep. pyogenes	1 (2.9%)
Seasonal allergies	1 (2.9%)

**Table 4:** Test Characteristics With POCUS Expert as the Standard and Kappa Coefficient Assessing Inter‐Rater Reliability

Test Characteristics	Values
Novice learners	7
Average novice scans submitted	4.57 (±1.32)
Median scan time (minutes)	7
Min–max; interquartile	3–43; 8
Sensitivity % (95% CI)	66.7 (9.4–99.1)
Specificity % (95% CI)	96.5 (82.2–99.9)
Positive likelihood ratio	19.3 (2.40–155.6)
Negative likelihood ratio	0.35 (0.07–1.17)
Positive predictive value % (95% CI)	67.1 (19.9–94.1)
Negative predictive value % 95% CI)	96.5 (84.9–99.2)
Accuracy % 95% CI)	93.7 (79.1–99.2)
Expert Cohen's kappa coefficient %	0.80 (80% agreement)

Min, minimum; Max, maximum.

For NLs' AI‐augmented interpretation, we found that the sensitivity, specificity, and accuracy were 66.7, 96.5, and 93.7%, respectively. NLs accurately diagnosed two of the three patients presenting with pneumonia. The third patient was determined to be indeterminate due to indistinct subpleural hypoechogenic consolidation in the presence of greater than three B‐lines. The positive LR was 19.3, and the negative LR was 0.35. Inter‐rater reliability between expert sonographers was high with a kappa coefficient of 0.8 (Table 4). Of the images reviewed for kappa by each expert, two discrepancies were resolved by a third expert reviewer.

The average quality rating overall was 2.94. We found minimal variability in quality rating when comparing left and right thorax, and anterior, lateral, or posterior sweeps (Table 5). All patients were able to be scanned in each sweep plane as shown in Figure 1.

**Table 5:** Image Quality Rating by Orientation. Below Average Represented Likert Rating 1–2, Average 3, and Above 4–5

Image Quality by Zone		Below Average	Average	Above Average	*n* = 32 Total Rating Average
Right thorax	AL	9 (25.7%)	13 (46.4%)	9 (31.0%)	3.03 (±0.18)
	LL	10 (28.6%)	9 (32.2%)	12 (41.4%)	3.06 (±0.23)
	PL	16 (45.7%)	6 (21.4%)	8 (27.6%)	2.70 (±0.22)
Total		35 (100.0%)	28 (100.0%)	29 (100.0%)	2.93 (±0.85)
Left thorax	AL	10 (29.4%)	11 (39.3%)	9 (30.0%)	2.93 (±0.21)
	LL	12 (35.3%)	7 (25.0%)	12 (40.0%)	3.03 (±0.24)
	PL	12 (35.3%)	10 35.7%)	9 (30.0%)	2.87 (±0.23)
Total		34 (100.0%)	28 (100.0%)	30 (100.0%)	2.95 (±0.62)

## Discussion

The use of lung POCUS has become a reliable diagnostic tool for assessing and managing patients presenting to the ED with respiratory complaints. For the diagnosis of community‐acquired pneumonia, lung POCUS has many benefits over the more commonly utilized CXR including avoiding exposure to ionizing radiation, lower cost, and higher sensitivity and specificity.^3^ A recent meta‐analysis by Yan et al concludes that lung POCUS should be considered as a first‐line imaging modality in the diagnosis of pediatric pneumonia.^14^ However, it is one of the more challenging examinations to interpret because it relies on visualizing and understanding artifacts, rather than anatomic structures for diagnostic recognition.^7^ The majority of studies to date evaluating the performance of traditional lung POCUS for pediatric community‐acquired pneumonia include highly trained or expert sonographers, thus limiting the generalizability of this method for diagnosis. In 2018, Correa et al offered a potential solution by suggesting an artificial neural network to detect the evidence of pneumonia infiltrates in US lung images as a foundation to improve the detection of pneumonia when compared to visual recognition performed by experts.^12^ Further advancement of these emerging algorithms in developing AI‐assisted software has created an opportunity to incorporate this technology in the clinical setting as shown in this study.

In our study, we sought to determine whether a machine‐assisted deep learning tool could augment the interpretation and accuracy of lung POCUS in pediatric patients presenting with respiratory complaints to a large tertiary care pediatric hospital. As a result, we found high specificity and diagnostic accuracy, but moderate‐to‐low sensitivity. The diagnostic accuracy of lung POCUS performed by NLs has been previously documented with overall sensitivity and specificity as high as 80 and 96% respectively, with a significant difference in the diagnostic accuracy for pneumonia between novice and advanced sonographers.^9^, ^16^ The sensitivity found in our study was similar or lower compared to prior studies.^6^, ^9^, ^16^, ^17^ It is possible that this result may be due to the small sample size of the study.

Typically, the pediatric lung POCUS examination requires six lung zones in each hemithorax with upper/lower anterior, lateral, and posterior views. While similar to adult‐based protocols, the pediatric approach offers a more robust assessment for the presence of pneumonia to investigate all lung fields since a large portion of pediatric pneumonia is found posteriorly.^8^, ^18^, ^19^ In this study, the machine‐assisted software offered a simplified three‐lung‐zone approach to enhance the efficiency while preserving accuracy and quality. We trained seven NLs with limited to no experience with lung POCUS on the current standard segmented approach, then subsequently introduced the software training, culminating with hands‐on education during the 1‐hour session. While a few of the NL had experience in using US for other applications such as peripheral IV placement and skin and soft tissue infections, all reported between 0 and 10 limited lung POCUS scans. Expert sonographers determined the overall image quality to be good and found the highest rating in the LL orientation, even though this was not significant in comparison to the other views. Interestingly, feedback from NLs suggested a preference of the PL orientation, likely due to its sweep length and ease of acquisition in patients who required distraction to cooperate with the examination, such as in a young infant (data not shown). This is important to note since the quality of the AI‐acquired images may also be dependent on transducer position and plane orientation. Nevertheless, the quality of the images in this study was consistent with other study findings as lung POCUS is one of the easier US applications to perform.^20^, ^21^

A total of 32 patients were evaluated; the majority of which were diagnosed with viral syndrome while 3 patients were diagnosed with pneumonia. Of these three, one was initially interpreted as indeterminate by the NL and one by an expert, while the second expert reviewing the study for agreement determined it to be positive based on pleural line abnormality and B‐lines (online supplemental Video 2). This discrepancy was finally resolved by the third expert reviewer who determined the interpretation as pneumonia. Of note, the other discrepancy resolved by the third reviewer in this study included image assessment determined to be normal after further evaluation. Independent of our evaluation, 63% of the patients, including those diagnosed with pneumonia, received a CXR. Pneumonia was detected in two of the patients receiving CXR, even though it is not the gold standard and may be inferior to POCUS evaluation. Additionally, CXR was not able to detect pneumonia in the third patient who was interpreted initially as indeterminate by the NLs' AI‐augmented interpretation and subsequently required three POCUS expert reviews. No patient required admission, and all were discharged in good condition. NLs completed close to five lung POCUS examinations on average within 7 minutes of scanning patients. This was consistent with the previous literature standard of 5 and 15 minutes.^9^ Factors that may have influenced this time include troubleshooting and managing the software at the bedside.

While patients in this pilot study were generalizable to the local population, the majority of patients were quite young which presented challenges commonly observed during POCUS evaluation. Despite these inherent difficulties, a machine‐assisted evaluation of the lung offered a rapid and efficient approach to image acquisition while preserving satisfactory image quality for interpretation and patient disposition.

The support of AI software for lung evaluation as described in this study underlies a potential vital role for this tool as the technology continues to advance. As shown in prior studies, AI can simplify tasks for LNs and promote rapid medical decision‐making, efficiency, and enhance the accuracy in diagnostic evaluation.^22^ The current standard interpretation of lung POCUS evaluation of pneumonia offers some challenges for experienced sonographers including optimized image acquisition, distribution of B‐lines, and pleural line characteristics. For NLs, these inherent challenges can limit accuracy of POCUS lung evaluation. Our study shows a potential solution for AI to support the innate challenges with lung US interpretation. As the need for, and utility of, this radiological method continues to grow, new innovations such as AI software and hardware may offer other solutions to improve the acquisition and diagnostic interpretation, particularly in novice sonographers. These data highlight the potential role of AI software in the future of POCUS lung evaluation where machine‐assisted interpretation can augment efficient and accurate diagnosis.

## Limitations

Since this was a small, single‐center study, the findings may not be generalizable to other practice environments. Additionally, the majority of patients scanned were younger and this age group can present unique challenges, which may limit our understanding of the machine–AI on other pediatric ages. Furthermore, only three patients had pneumonia, so it is unclear whether the results would be consistent in a population with a higher disease prevalence. The low prevalence of pneumonia in this study does not reflect our institution's ED population but rather the small scale of this study to understand how AI can augment image acquisition and interpretation. Additionally, our inclusion criteria may have not been specific enough to capture the true prevalence which is higher. While NLs had ongoing diverse extensive medical training and some prior experience with US, it is unclear whether NLs without similar prior medical education would perform similarly. Even though the PL orientation is important to clinical assessment, it is limited to patients restricted to the supine position such as in critical care patients. However, the LL and AL views are sufficient similarly to the previously described eight‐zone protocol which offers a generalizable evaluation of lung pathology.^8^, ^18^ Lastly, confounding factors such as body habitus, patient cooperation, discomfort, and troubleshooting software glitches may all affect the performance of the software and will need to be controlled and further explained in future studies.

## Conclusion

In this small pilot study, we found high accuracy and specificity for NLs' AI‐assisted lung POCUS interpretations when compared to expert diagnosis. While the study is limited in its scope of generalizability, the findings support the emerging use of AI and deep learning algorithms to assist lung POCUS image acquisition and interpretation. Future studies to further improve the performance of AI in POCUS and its application to various respiratory presentations will need to be addressed.

## Supporting information


**Video S1** Supplementary Video

**Video S2** Supplementary Video
